# The Effect of Inhaled Chromium on Different Exhaled Breath Condensate Biomarkers among Chrome-Plating Workers

**DOI:** 10.1289/ehp.8506

**Published:** 2005-12-08

**Authors:** Andrea Caglieri, Matteo Goldoni, Olga Acampa, Roberta Andreoli, Maria Vittoria Vettori, Massimo Corradi, Pietro Apostoli, Antonio Mutti

**Affiliations:** 1 Laboratory of Industrial Toxicology, Department of Clinical Medicine, Nephrology and Health Sciences, University of Parma, Italy; 2 National Institute of Occupational Safety and Prevention, Research Centre at the University of Parma, Parma, Italy; 3 Laboratory of Industrial Hygiene, Department of Experimental and Applied Medicine, University of Brescia, Brescia, Italy

**Keywords:** chromium, exhaled breath condensate, hydrogen peroxide, lung, malondialdehyde

## Abstract

Chromium is corrosive, cytotoxic, and carcinogenic for humans and can induce acute and chronic lung tissue toxicity. The aim of this study was to investigate Cr levels in exhaled breath condensate (EBC) of workers exposed to Cr(VI) and to assess their relationship with biochemical changes in the airways by analyzing EBC biomarkers of oxidative stress, namely, hydrogen peroxide (H_2_O_2_) and malondialdehyde (MDA). EBC samples were collected from 24 chrome-plating workers employed in a chrome-plating plant both before and after the Friday work shift and before the work shift on the following Monday. Cr-EBC levels increased from the beginning (5.3 μg/L) to the end of Friday (6.4 μg/L) but were considerably lower on Monday morning (2.8 μg/L). A similar trend was observed for H_2_O_2_-EBC levels (which increased from 0.36 μM to 0.59 μM on Friday and were 0.19 μM on Monday morning) and MDA-EBC levels (which increased from 8.2 nM to 9.7 nM on Friday and were 6.6 nM on Monday). Cr-EBC levels correlated with those of H_2_O_2_-EBC (*r* = 0.54, *p* < 0.01) and MDA-EBC (*r* = 0.59, *p* < 0.01), as well as with urinary Cr levels (*r* = 0.25, *p* < 0.05). The results of this study demonstrate that EBC is a suitable matrix that can be used to investigate both Cr levels and biomarkers of free radical production sampling the epithelial-lining fluid of workers exposed to Cr(VI).

Chromium is a transition element occurring in the environment (soil, rocks, plants, dust, and gases), primarily in the elemental, trivalent [Cr(III)], and hexavalent [Cr(VI)] oxidation states. Both Cr(III) and Cr(VI) are environmentally stable, and their toxicologic profiles are well known; Cr(III) has limited toxicologic properties [De Flora et al. 1990; International Agency for Research on Cancer (IARC) 1990] and is considered to be an essential trace metal in humans (Anderson 1981), whereas various Cr(VI) compounds are considered to be human carcinogens [Agency for Toxic Substances and Disease Registry (ATSDR) 2000; De Flora 2000; Ding and Shi 2002; IARC 1990; Kawanishi et al. 2002; O’Brien et al. 2003], are known to induce both acute and chronic toxic effects (ATSDR 2000), and are of the greatest occupational and environmental health concern.

The respiratory tract is the main target organ of Cr(VI) toxicity associated with both acute (short-term) and chronic (long-term) inhalation (ATSDR 2000; De Flora 2000): acute exposure may cause shortness of breath, coughing, and wheezing (Sobaszek et al. 2000). Chronic exposure leads to ulcerations and perforations of the nasal septum, chronic bronchitis, decreased pulmonary function, pneumonia, and other respiratory effects (Bradshaw et al. 1998). On the basis of experimental and epidemiologic evidence (De Flora 2000; Gibb et al. 2000; Luippold et al. 2005; Park et al. 2004), IARC has classified Cr(VI) as a class 1 carcinogen (recognized human carcinogen).

Cr(VI) compounds are used in several industrial applications (chrome plating, welding inox steel and other special steels, painting, leather tanning, and wood preserving). Occupational exposure mainly occurs by inhalation, but it may involve the gastrointestinal tract and skin (De Flora 2000). Therefore, the respiratory tract is the primary target organ for Cr(VI) compounds. Experimental work on the rat showed that lung accumulation of Cr(VI) can be observed even after intravenous administration (Mutti et al. 1979). The mechanism of Cr(VI) cytotoxicity is not completely understood, but several studies have shown that Cr(VI) compounds induce oxidative stress, DNA damage, apoptotic cell death, and altered gene expression (Bagchi et al. 2002; Wise et al. 2002; Zhitkovich 2005). The reduction in Cr(VI) levels induced by redox-active enzymes and small molecules generates intermediate unstable states, such as Cr(V) or Cr(IV), that may mediate the formation of free hydroxyl, thiyl, ascorbate, and carbon-based radicals (Ding and Shi 2002; Levina and Lay 2005; O’Brien et al. 2003) that are capable of damaging macromolecular targets, such as DNA (Stohs et al. 2001). Interestingly, although Cr(III) reacts with DNA and proteins, it is unable to cross cell membranes. The opposite occurs for Cr(VI) species, which do not react with nucleophilic targets but can easily cross cell membrane through anion channels. Once inside the cell, Cr(VI) is rapidly reduced to the trivalent state, and Cr(III) then interacts with cell proteins and DNA (Levina and Lay 2005).

Improved work areas, procedures, and hygiene measures have minimized occupational exposure to Cr(VI) compounds and led to a reduction in traditional adverse effects on the lung, such as tracheobronchitis or pneumonia; however, long-term Cr(VI) exposure may still cause airway disorders, including airway irritation, sensitization, and lung cancer. Sensitive tests are therefore needed to evaluate early biochemical changes that occur in the respiratory tract after Cr(VI) exposure. Furthermore, because the respiratory tract is the primary route of exposure to Cr(VI), the quantification of biomarkers of free radical production at the target organ level could improve the sensitivity and specificity of putative biomarkers.

We have recently shown that exhaled breath condensate (EBC), a fluid formed as a result of the cooling of expired air, is a suitable matrix not only for assessing the biomarkers of oxidative stress in exposed workers [malondialdehyde (MDA)] but also for quantifying the levels of some pneumotoxic substances in the lung, in particular, cobalt (Goldoni et al. 2004). The synergistic effect of tungsten to power the lipid peroxidation caused by cobalt has also been demonstrated (Goldoni et al. 2004).

The aim of the present study was to investigate Cr levels in the EBC of workers employed in the chrome-plating industry and to assess early biochemical changes in the airways by analyzing EBC biomarkers of oxidative stress, such as hydrogen peroxide (H_2_O_2_) and MDA.

## Materials and Methods

### Subjects.

Table 1 shows the demographic and clinical characteristics of the 24 chrome-plating workers recruited in the study, each of whom carried out various tasks during the same work day. Four of these subjects have a history of light smoking status (< 10 cigarettes/day, corresponding to < 10 packs/year), but we did not find any differences in exhaled biomarker levels between smoking and nonsmoking workers. All of the workforce volunteered to participate in this study, which took place during paid work hours. The “healthy worker effect” cannot be ruled out, owing to preemployment selection procedures. However, all of these workers underwent medical health surveillance, and all were asymptomatic and presumably healthy. The control group consisted of 25 healthy adult volunteers with no significant history of lung disease and who were not occupationally exposed to Cr. The workers also exhibited normal spirometric indices and did not report any significant previous or current respiratory diseases. None of them had any symptoms of acute respiratory illness during the 4 weeks preceding the study. We found no differences between male and female control subjects for the studied biomarkers.

### Study design.

Before enrollment, the subjects completed a short questionnaire concerning their current and previous medical history and underwent a spirometric examination. The EBC and urine samples of the workers were assessed three times: *a*) before the work shift on Friday (*T*_0_); *b*) at the end of the same shift, after 8 hr of work (*T*_1_); and *c*) before exposure on the following Monday (*T*_2_). EBC and urine samples from the controls were collected in our laboratory during a normal work day, and a subgroup of 10 subjects repeated the procedures in the office area of the work-place in order to exclude any contamination by the office environmental air.

All of the subjects gave their written informed consent to the procedures, which were approved by our local ethics committee. The biologic material was sampled as described in the Declaration of Helsinki (World Medical Association 2002).

### Spirometric measurements.

Spirometry was performed using a pneumotachograph (Koko Spirometer, Sensormedics, Milan, Italy). Mean forced expiratory volume in 1 sec (FEV_1_) and forced vital capacity (FVC) were obtained from the three best acceptable test values of lung function, as recommended by the American Thoracic Society (1995).

### Environmental measurements.

Ambient monitoring was carried out by personal samplers. Briefly, airborne particulate was collected on polyvinyl chloride membrane filters (5.0 μm porosity, 25 mm diameter) according to National Institute of Occupational Safety and Health (NIOSH) protocol 7300 (NIOSH 2003a) at a constant flow of 3 L/min for a period ranging from 90 to 150 min in the morning or afternoon of the Friday work shift. The membranes were weighed in a thermo-hygrometrically conditioned cabinet using a precision balance with a sensitivity of 0.01 mg and dissolved in concentrated hyperpure nitric acid (70%), and the solution was diluted with ultrapure water. The analytical blank was obtained from virgin membranes. Total Cr was analyzed by means of electrothermal atomic absorption spectroscopy (ETAAS) with Zeeman background correction following UNICHIM (Italian Association for Unification in the Sector of Chemical Industry) method 886 (UNICHIM 1995), and expressed as micrograms per cubic meter. To validate the precision and the sensitivity of our method, we used the standard addition method using a certified Cr(VI) standard solution (Fluka, Milan, Italy). The amount of soluble hexavalent Cr in the ambient air was estimated following NIOSH protocol 7703 (NIOSH 2003b) after air collection using stationary samplers in the work rooms and office of the plant.

Airborne concentrations of total Cr for the Friday work shift were 8.8 (2.6) μg/m^3^ [GM (GSD)] in the morning and 5.0 (2.5) μg/m^3^ in the afternoon, and the weighted average was 7.8 (2.3) μg/m^3^. Workers were significantly more exposed in the morning (*p* < 0.05) than during the afternoon half-shift, whereas there were no significant differences in the collection times between morning and afternoon. Soluble Cr(VI) was about 70% of the total Cr monitored in the environment (data not shown); therefore, all measured Cr samples were below the limit proposed by the American Conference of Governmental Industrial Hygienists (ACGIH 1999) for water-soluble Cr(VI) organic compounds (50 μg/m^3^).

### EBC collection.

**Figure 1:**
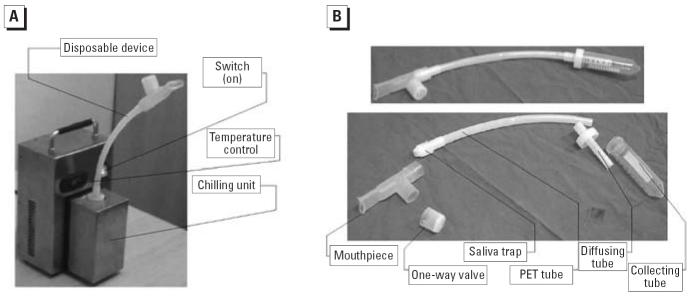
(*A*) TURBO-DECCS condenser. (*B*) DECCS-system disposable device. PET, polyethylene.

EBC was collected using a new portable TURBO-DECCS condenser (transportable unit for research on biomarkers obtained from disposable exhaled condensate collection systems; ItalChill, Parma, Italy; Figure 1A) specifically designed to collect EBC in clinical and work settings. The condenser has a refrigerating system (TURBO) that thermostatically controls the working temperature, and a disposable respiratory system (DECCS) that consists of a mouthpiece connected to a one-way aspiration valve and (through a tube with a special stopper) an EBC collection test tube at the end (Figure 1B). On the basis of preliminary data (not shown), we chose a condensation temperature of –5°C (Goldoni et al. 2005), a good compromise between the yield in terms of volume for multianalyses and detectable concentrations of the selected analytes.

The subjects were asked to breathe tidally through the mouthpiece for 15 min, while sitting comfortably in the workplace office where the level of total Cr was < 0.1 μg/m^3^ (workers) or in the laboratory office (controls). They were instructed to form a complete seal around the mouthpiece with their mouths, which had to be kept dry by periodically swallowing excess saliva; they were also asked to rinse their mouths thoroughly before the maneuver and every 5 min during the test. To prevent any contamination from the skin, the subjects were asked to wash their hands before EBC and urine collection and to wear disposable latex gloves during the collecting procedures. EBC sample volume was accurately measured with a calibrated pipette, and salivary contamination was excluded by means of the colorimetric detection of alpha-amylase activity (Infinity amylase reagent; Sigma, Milan, Italy). Moreover, we measured the levels of Cr in saliva of five workers and found a maximum salivary Cr level of about 10 times higher than the corresponding EBC value, not sufficient to justify salivary contamination, given that the limit of detection (LOD) of the amylase kit was much lower (between 1/10,000 and 1/5,000) than the amylase activity measured in saliva. Finally, the EBC samples were transported in dry ice to the laboratory and stored at −80°C until analysis.

### Analysis of Cr in urine and EBC.

Urinary Cr (Cr-U) was measured by ETAAS and expressed as a function of creatinine. Urinary overcorrection was excluded because none of the subjects presented creatinine levels < 30 mg/L or > 300 mg/L. The LOD was 0.05 μg/L, and a certified standard (Fluka) was repeated every 10 samples.

The ETAAS analysis protocol was applied to the EBC samples, and the results were confirmed by inductively coupled plasma–mass spectrometry (ICP-MS) in a separate laboratory, with an interlaboratory regression coefficient of 0.95 and an average deviation between samples of about 10% (data not shown). The LOD was 0.05 μg/L.

### H_2_O_2_-EBC measurements.

H_2_O_2_-EBC was measured as previously described (Corradi et al. 2002) using a commercial kit (Amplex Red hydrogen peroxide assay kit; Molecular Probes, Eugene, OR, USA) with an LOD of 0.01 μM. The analyses were made 2–3 days after EBC collection. Samples were stored at −80°C up to 3 days at the most (Goldoni et al. 2005).

### MDA-EBC measurements.

MDA-EBC was measured by tandem liquid chromatography–mass spectrometry as previously described by Andreoli et al. (2003), with an LOD of 1 nM.

### Statistical analysis.

The data were analyzed using SPSS 13.0 (SPSS, Chicago, IL, USA) and PRISM 3.0 (Graphpad Software, San Diego, CA, USA). The measured parameters showed a log-normal distribution (Shapiro-Wilk test) for all the considered groups of subjects; therefore, the data were log-transformed for all of the statistical tests, and the results were expressed as the geometric mean (GM) and geometric SD (GSD). Median values and interquartile ranges are also reported. We analyzed log-transformed data using repeated-measures analysis of variance (ANOVA) followed by Tukey’s multiple comparisons or a paired Student *t*-test in order to assess the differences within the workers’ group at different times. One-way ANOVA, followed by Dunnett’s multiple comparisons, was used to assess the differences between the workers and controls. Pearson’s *r* was used to assess the correlations between variables in the log-scale. The normality is required for all parametric comparison tests, whereas for tests assessing differences between repeated measures in dependent samples, a normal distribution is required for those differences. A log-transformation of our data was necessary to satisfy both the above requirements. Moreover, such a log-transformation was necessary to limit the undue influence of the highest values on the *r*and *p*-values. A *p*-value of < 0.05 (two-tailed) was considered significant for all of the tests.

## Results

**Figure 2:**
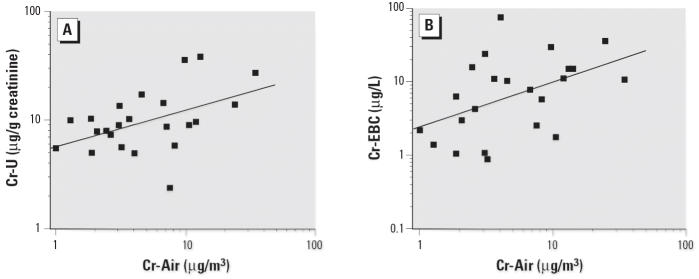
Correlations, with linear regression lines and Pearson’s *r,* between environmental airborne Cr (Cr-Air) and Cr-U (*A; r* = 0.48, *p* < 0.05) and between Cr-Air and Cr-EBC (*B; r =* 0.47, *p* < 0.05).

Ambient Cr levels sampled during the afternoon moderately correlated with Cr-U (*r* = 0.48, *p* < 0.05) and Cr-EBC at *T*_1_ (*r* = 0.47, *p* < 0.05), as shown in Figure 2.

Table 2 shows the Cr-U and Cr-EBC levels at the different time points. Compared with the controls, the exposed workers had higher levels of both at all of the time points (except for a few Cr-EBC levels at *T*_2_) (*p* < 0.01). Cr-U levels were higher at *T*_1_ than at *T*_0_ (*p* < 0.05) and *T*_2_ (*p* < 0.01), and the difference between *T*_0_ and *T*_2_ was also significant (*p* < 0.01). Cr-EBC levels were higher at *T*_0_ and *T*_1_ than at *T*_2_ (*p* < 0.05 and *p* < 0.01, respectively), whereas no difference was observed between *T*_0_ and *T*_1_.

Workers’ H_2_O_2_-EBC levels were higher than those of the controls at *T*_0_ (*p* < 0.05) and *T*_1_ (*p* < 0.01), and there was a significant difference between *T*_2_ and both *T*_0_ and *T*_1_ (*p* < 0.05 and *p* < 0.01, respectively); the difference between *T*_0_ and *T*_1_ was not significant (Table 2).

The MDA-EBC levels of workers were not significantly higher than those of the controls at every time point, although 12 of 24 workers (whose median of Cr-EBC was 11 μg/L) presented MDA levels above the 95% confidence interval (CI) upper limit for controls (10 nM) at *T*_1_. The other workers with MDA-EBC < 10 nM showed a median of 2.5 μg/L of Cr-EBC. The only significant difference in MDA-EBC was between *T*_1_ and *T*_2_ (*p* < 0.01, Table 2).

**Figure 3:**
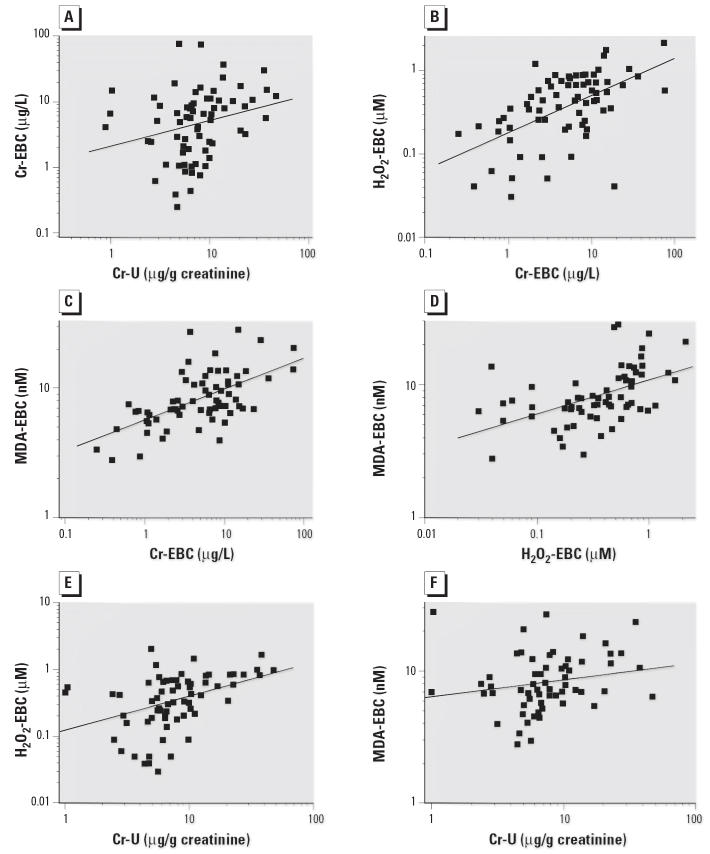
Correlations between variables, with linear regression lines, Pearson’s *r*, and significance. (*A*) Cr-U versus Cr-EBC (*r* = 0.25, *p* < 0.05). (*B*) Cr-EBC versus H_2_O_2_-EBC (*r* = 0.54, *p* < 0.01). (*C*) Cr-EBC versus MDA-EBC (*r* = 0.59, *p* < 0.01). (*D*) H_2_O_2_-EBC versus MDA-EBC (*r* = 0.49, *p* < 0.01). (*E*) Cr-U versus H_2_O_2_ EBC (*r* = 0.41, *p* < 0.01). (*F*) Cr-U versus MDA-EBC (*r* = 0.19, not significant).

All nonparametric tests gave results consistent with those observed using their parametric counterpart on log-transformed values. Cr-U and Cr-EBC levels presented a weak-to-moderate correlation at all time points (*r* = 0.25, *p* < 0.05; Figure 3A). Cr-EBC and H_2_O_2_-EBC correlated closely (*r* = 0.54, *p* < 0.01; Figure 3B), particularly at *T*_1_ (*r* = 0.72, *p* < 0.01; data not shown); Cr-EBC also correlated with MDA-EBC (*r* = 0.59, *p* < 0.01; Figure 3C). H_2_O_2_ and MDA were positively correlated with each other (*r* = 0.49, *p* < 0.01; Figure 3D).

There was also a correlation between H_2_O_2_-EBC and Cr-U levels—although it was lower than the correlation between Cr-EBC and H_2_O_2_-EBC (*r* = 0.41, *p* < 0.01; Figure 3E)—whereas the correlation between Cr-U and MDA-EBC was not significant (Figure 3F). The significance of all the correlations was confirmed by the Spearman nonparametric correlation test.

We observed no statistically significant differences in EBC-collected volumes between exposed workers (mean ± SD, 1,545 ± 255 μL at *T*_0_, 1,460 ± 270 μL at *T*_1_, and 1,570 ± 280 μL at *T*_2_) and controls (1,585 ± 240 μL). The exhaled markers did not correlate with total condensed volume, and because there were no statistical differences between the total EBC volumes at the different time points, total condensed volume was excluded from the analysis. The total expired air was not measured because, as already demonstrated by Gessner et al. (2001), the total volume of expired air is highly correlated (*r* = 0.95) with the total volume of EBC.

## Discussion

Blood and urine are currently used for the biologic monitoring of workers exposed to Cr (Franchini and Mutti 1988; Mutti et al. 1984, 1985), and thus represent the gold standard matrices for the assessment of exposure. However, they provide little information concerning lung levels, which are not necessarily related to absorbed dose, but are certainly responsible for local inflammation on the respiratory tract. EBC analysis is a novel approach to biologic monitoring, enabling us to quantify both internal dose levels of inhaled metals and to assess epithelial-lining fluid levels of biomarkers of free radical production. The results of our study show that EBC is a suitable medium for assessing biomarkers of inflammation (i.e., oxidative stress) and that it can be used to investigate lung tissue levels and biomarkers of effect in workers exposed to Cr(VI). However, future studies are needed to look at the quantitative relationship between Cr concentration in air and EBC levels as well as the association with relevant clinical outcome to assess health effects, such as spirometry, or methacholine reactivity, and so forth.

Our study was designed to assess biologic markers at different sampling times in order to evaluate the consequences of long-term and daily lung exposure. Cr-EBC and Cr-U levels were much higher in exposed subjects than in controls at all time points. Furthermore, the two variables were only slightly positively correlated with each other, such a weak-to-moderate correlation being possibly explained by the relatively complex kinetics of Cr (long-life kinetic components inside the body, in particular after chronic exposure) and the presence of other absorption routes (e.g., dermal).

Owing to their moderate correlation with Cr levels in ambient air, both Cr-EBC and Cr-U can be considered biomarkers of exposure. However, further work is needed for a full validation of Cr-EBC as a biomarker of exposure and before this metric can be used independently to assess exposure or health risk.

Looking at the different correlations between variables, Cr-U and Cr-EBC levels seem to provide different information: there were weak or no correlations between Cr-U and biomarkers of free radical production, whereas Cr-EBC concentrations correlated with higher and significant *r-*values with both MDA and H_2_O_2_ levels in EBC, thus suggesting that they may be more representative of the lung dose responsible for local free radical production.

We assessed inflammation in the lung after Cr exposure, relying on biomarkers of oxidative stress, which may be sensitive end points for evaluating early biochemical changes in the airways. This approach will probably overcome the limitations of traditional spirometric tests, which often indicate late and frequently irreversible lung dysfunction.

Analyses of MDA and H_2_O_2_ levels in EBC have shown that they are reliable indices of airway oxidative stress (Corradi et al. 2003; Forteza et al. 2005), and they may be of some help in assessing and understanding the underlying mechanisms of metal-induced carcinogenesis. Accumulated evidence suggests that the oxidative stress caused by an imbalance of cellular free radical generation and anti-oxidative defense plays an important role in metal-induced cell responses and carcinogenesis (Desoize 2003; Sorensen et al. 2005). Although MDA and H_2_O_2_ levels were in the normal range after 2 days away from work, H_2_O_2_ levels increased to above-normal values after a weekly and a daily Cr exposure, whereas 50% of workers (the most exposed) showed MDA levels higher than the 95% CI upper limit of control MDA levels at the end of the work shift. However, we speculate that further influencing factors, such as individual susceptibility to Cr, may modify MDA levels. As in the case of the toxic effect of cigarette smoke on the lungs, it cannot be excluded that an intermittent and repeated inhaled Cr stimulus may also be responsible for lung damage. It is worth noting that the increased H_2_O_2_ levels observed in EBC of our workers at the end of a work day were similar to those reported by Gerritsen et al. (2005) in chronic obstructive pulmonary disease patients during exacerbations.

Unlike their H_2_O_2_ levels, the workers’ MDA-EBC levels did not increase after a day’s exposure, which suggests that H_2_O_2_ may be a more sensitive marker of acute lung free radical production in more highly Cr-exposed workers. However, MDA-EBC and H_2_O_2_-EBC were positively correlated, thus confirming the idea that the biologic induction of H_2_O_2_ (a marker of inflammatory processes) may increase *in vivo* oxidative stress and lipid peroxidation products.

Because there is a good correlation between Cr levels and airway free radical production and because many studies have identified Cr(VI) as the most toxic form of Cr, we are carrying out further studies on the characterization of Cr species in EBC samples after exposure to Cr(VI).

Finally, to explain the interindividual variability (e.g., workers with an anomalous trend compared with the average), it should be considered that different jobs and tasks in a chrome-plating plant may imply exposure to different Cr species. Different physicochemical properties, in terms of valence and solubility, may imply different kinetic and dynamic properties, thus accounting for a different behavior of lining fluid (EBC) compared with urine, whose Cr content is only justified by absorption of soluble Cr(VI).

In conclusion, EBC can be considered a promising medium for investigating both long-term and recent Cr exposure at target tissue level and, together with biomarkers of free radical production, it can provide insights into the oxidative lung interactions between pulmonary tissue and pneumotoxic metals occurring in exposed workers.

## Figures and Tables

**Table 1:** Demographic and clinical data of subjects participating in this study (mean ± SD).

	Controls	Chrome-plating workers
No.of subjects	25	24
Sex (male/female)	13/12	24/0
Age (years)	31.0 ± 6.7	37.7 ± 6.3
Smokers/ex-/non^a^	0/5/20	4/2/18
FVC (% predicted)	108 ± 10.4	89.8 ± 10.7
FEV_1_ (% predicted)	103.7 ± 11.4	91.8 ± 12.0
FEV_1_:FVC	81.9 ± 6.3	83.1 ± 7.5

a Smokers/ex-smokers/nonsmokers.

**Table 2:** Cr-U, Cr-EBC, H_2_O_2_-EBC, and MDA-EBC levels of controls and of workers at different sampling times.

	Cr-U (μg/g creatinine)	Cr-EBC (μg/L)	H_2_O_2_-EBC (μM)	MDA-EBC (nM)
Controls				
GM (GSD)	0.25 (2.3)	0.28 (2.6)	0.09 (1.6)	6.0 (1.5)
Median (25th–75th percentile)	0.24 (0.09–0.45)	0.25 (0.1–0.44)	0.12 (0.05–0.2)	6.5 (5.0–8.2)
Workers (*T*_0_)				
GM (GSD)	8.2 (2.1)**	5.3 (2.9)**	0.36 (2.4)*	8.2 (1.5)
Median (25th–75th percentile)	7.9 (6.2–10.8)	7.3 (2.3–9.2)	0.44 (0.22–0.61)	7.7 (6.6–11.5)
Workers (*T*_1_)				
GM (GSD)	9.4 (2.1)**^,#^	6.4 (3.4)**	0.59 (2.0)**	9.7 (1.7)
Median (25th–75th percentile)	9.0 (5.6–13.4)	7.8 (2.1–15.2)	0.69 (0.42–0.86)	10.7 (6.9–13.4)
Workers (*T*_2_)				
GM (GSD)	5.7 (2.2)**^,##,†^	2.8 (3.2)**,#,†	0.19 (2.6)#,†	6.6 (1.5)†
Median (25th–75th percentile)	5.6 (3.1–6.7)	4.0 (1.1–6.6)	0.21 (0.09–0.35)	6.8 (5.3–8.0)

* *p* < 0.05 and

** *p* < 0.01 compared with controls.

# *p* < 0.05 and

## *p* < 0.01 compared with *T*_0_.

† *p* < 0.01 compared with *T*_1_.

## References

[b1-ehp0114-000542] ACGIH 1999. Chromium. Documentation of the Threshold Limit Values and Biological Exposure Indices. Cincinnati, OH:American Conference of Governmental Industrial Hygienists.

[b2-ehp0114-000542] American Thoracic Society (1995). Standardization of spirometry, 1994 update. Am J Respir Crit Care Med.

[b3-ehp0114-000542] Anderson RA (1981). Nutritional role of chromium. Sci Tot Environ.

[b4-ehp0114-000542] Andreoli R, Manini P, Corradi M, Mutti A, Niessen WM (2003). Determination of patterns of biologically relevant aldehydes in exhaled breath condensate of healthy subjects by liquid chromatography/atmospheric chemical ionization tandem mass spectrometry. Rapid Commun Mass Spectrom.

[b5-ehp0114-000542] ATSDR 2000. Final Report of Toxicological Profile for Chromium. NTIS Accession No. PB2000-108022. Atlanta, GA:Agency for Toxic substances and Disease Registry. 24049864

[b6-ehp0114-000542] Bagchi D, Stohs SJ, Downs BW, Bagchi M, Preuss HG (2002). Cytotoxicity and oxidative mechanisms of different forms of chromium. Toxicology.

[b7-ehp0114-000542] Bradshaw LM, Fishwick D, Slater T, Pearce N (1998). Chronic bronchitis, work related respiratory symptoms, and pulmonary function in welders in New Zealand. Occup Environ Med.

[b8-ehp0114-000542] Corradi M, Alinovi R, Goldoni M, Vettori MV, Folesani G, Mozzoni P (2002). Biomarkers of oxidative stress after controlled human exposure to ozone. Toxicol Lett.

[b9-ehp0114-000542] Corradi M, Rubinstein I, Andreoli R, Manini P, Caglieri A, Poli D (2003). Aldehydes in exhaled breath condensate of patients with chronic obstructive pulmonary disease. Am J Respir Crit Care Med.

[b10-ehp0114-000542] De Flora S (2000). Threshold mechanisms and site specificity in chromium(VI) carcinogenesis. Carcinogenesis.

[b11-ehp0114-000542] De Flora S, Bagnasco M, Serra D, Zanacchi P (1990). Genotoxicity of chromium compounds. A review. Mutat Res.

[b12-ehp0114-000542] Desoize B (2003). Metals and metal compounds in carcinogenesis. In Vivo.

[b13-ehp0114-000542] Ding M, Shi X (2002). Molecular mechanisms of Cr(VI)-induced carcinogenesis. Mol Cell Biochem.

[b14-ehp0114-000542] Forteza R, Salathe M, Miot F, Forteza R, Conner GE (2005). Regulated hydrogen peroxide production by Duox in human airway epithelial cells. Am J Respir Cell Mol Biol.

[b15-ehp0114-000542] Franchini I, Mutti A (1988). Selected toxicological aspects of chromium(VI) compounds. Sci Total Environ.

[b16-ehp0114-000542] Gerritsen WB, Asin J, Zanen P, van den Bosch JM, Haas FJ (2005). Markers of inflammation and oxidative stress in exacerbated chronic obstructive pulmonary disease patients. Respir Med.

[b17-ehp0114-000542] Gessner C, Kuhn H, Seyfarth HJ, Pankau H, Winkler J, Schauer J (2001). Factors influencing breath condensate volume. Pneumologie.

[b18-ehp0114-000542] Gibb HJ, Lees PSJ, Pinsky PF, Rooney BC (2000). Lung cancer among workers in chromium chemical production. Am J Ind Med.

[b19-ehp0114-000542] Goldoni M Caglieri A Andreoli R Poli D Manini P Vettori MV 2005 Influence of condensation temperature on selected exhaled breath parameters BMC Pulm Med 5 1010.1186/1471-2466-5-10. 16137323 10.1186/1471-2466-5-10 PMC1236937

[b20-ehp0114-000542] Goldoni M, Catalani S, De Palma G, Manini P, Acampa O, Corradi M (2004). Exhaled breath condensate as a suitable matrix to assess lung dose and effects in workers exposed to cobalt and tungsten. Environ Health Perspect.

[b21-ehp0114-000542] IARC (International Agency for Research on Cancer) (1990). Chromium, Nickel and Welding. IARC Monogr Eval Carcinog Risk Hum.

[b22-ehp0114-000542] Kawanishi S, Hiraku Y, Murata M, Oikawa S (2002). The role of metals in site-specific DNA damage with reference to carcinogenesis. Free Radic Biol Med.

[b23-ehp0114-000542] Levina A, Lay PA (2005). Mechanistic studies of relevance to the biological activities of chromium. Coord Chem Rev.

[b24-ehp0114-000542] Luippold RS, Mundt KA, Dell LD, Birk T (2005). Low-level hexavalent chromium exposure and rate of mortality among US chromate production employees. J Occup Environ Med.

[b25-ehp0114-000542] Mutti A, Cavatorta A, Borghi L, Canali M, Giaroli C, Franchini I (1979). Distribution and urinary excretion of chromium. Studies on rats after administration of single and repeated doses of potassium dichromate. Med Lav.

[b26-ehp0114-000542] Mutti A, Lucertini S, Valcavi P, Neri TM, Fornari M, Alinovi R (1985). Urinary excretion of brush-border antigen revealed by monoclonal antibody: early indicator of toxic nephropathy. Lancet.

[b27-ehp0114-000542] Mutti A, Pedroni C, Arfini G, Franchini I, Minoia C, Micoli G (1984). Biological monitoring of occupational exposure to different chromium compounds at various valency states. Int J Environ Anal Chem.

[b28-ehp0114-000542] NIOSH (National Institute of Occupational Safety and Health) 2003a. NIOSH Manual of Analytical Methods (NMAM). 4th ed. Method 7300: Elements by ICP (Nitric/Perchloric Acid Ashing). Available: http://www.cdc.gov/niosh/nmam/pdfs/7300.pdf [accessed 18 October 2005].

[b29-ehp0114-000542] NIOSH (National Institute of Occupational Safety and Health) 2003b. NIOSH Method 7703: Hexavalent Chromium by Field-Portable Spectrophotometry. Available: http://www.cdc.gov/niosh/nmam/pdfs/7703.pdf [accessed 10 July 2005].

[b30-ehp0114-000542] O’Brien TJ, Ceryak S, Patierno SR (2003). Complexities of chromium carcinogenesis: role of cellular response, repair and recovery mechanisms. Mut Res.

[b31-ehp0114-000542] Park RM, Bena JF, Stayner LT, Smith RJ, Gibb HJ, Lees PS (2004). Hexavalent chromium and lung cancer in the chromate industry: a quantitative risk assessment. Risk Anal.

[b32-ehp0114-000542] Sobaszek A, Boulenguez C, Frimat P, Robin H, Haguenoer JM, Edme JL (2000). Acute respiratory effects of exposure to stainless steel and mild steel welding fumes. J Occup Environ Med.

[b33-ehp0114-000542] Sorensen M, Schins RP, Hertel O, Loft S (2005). Transition metals in personal samples of PM_2.5_ and oxidative stress in human volunteers. Cancer Epidemiol Biomarkers Prev.

[b34-ehp0114-000542] Stohs SJ, Bagchi D, Hassoun E, Bagchi M (2001). Oxidative mechanisms in the toxicity of chromium and cadmium ions. J Environ Pathol Toxicol Oncol.

[b35-ehp0114-000542] UNICHIM (Italian Association for Unification in the Sector of Chemical Industry) 1995. 886: Ambienti di Lavoro—Determinazione di Nichel, Manganese e Cromo nei Fumi di Saldatura—Metodo con Spettrometro ad Assorbimento Atomico Dotato di Fornetto di Grafite. Available: http://www.unichim.it/htm/pagine_pubblicazioni/pagina_iniziale_pubblicazioni.htm [accessed 15 October 2005].

[b36-ehp0114-000542] Wise JP, Wise SS, Little JE (2002). The cytotoxicity and genotoxicity of particulate and soluble hexavalent chromium in human lung cells. Mut Res.

[b37-ehp0114-000542] World Medical Association 2002. World Medical Association Declaration of Helsinki. Ethical Principles for Medical Research Involving Human Subjects. Ferney-Voltaire, France:World Medical Association. Available: http://www.wma.net/e/policy/pdf/17c.pdf [accessed 15 April 2005].

[b38-ehp0114-000542] Zhitkovich A (2005). Importance of chromium-DNA adducts in mutagenicity and toxicity of chromium(VI). Chem Res Toxicol.

